# Efficacy of virtual reality interventions in reducing preoperative anxiety in pediatric patients undergoing general anesthesia: a systematic review and meta-analysis

**DOI:** 10.7717/peerj.21123

**Published:** 2026-04-22

**Authors:** Jinzhuo Song, Yuming Tu, Qinyi Chen, Rong Bao, Xihong Ye

**Affiliations:** 1 Hubei University of Arts and Science, Xiangyang, China; 2 Department of Anesthesiology, Xiangyang Central Hospital, Affiliated Hospital of Hubei University of Arts and Science, Xiangyang, China

**Keywords:** Virtual reality, Preoperative anxiety, Pediatric patients, General anesthesia, Meta-analysis

## Abstract

**Objective:**

To evaluate the effectiveness of virtual reality (VR) interventions in reducing preoperative anxiety in pediatric patients undergoing general anesthesia, and provide evidence to inform clinical decision-making.

**Methods:**

A comprehensive search was performed across PubMed, Embase, and the Cochrane Library, covering publications from inception to June 7, 2025. The study included randomized clinical trials (RCTs) of VR-based interventions (both immersive and non-immersive) compared to standard treatments or placebo controls. Studies assessing primary outcomes such as anxiety levels before induction, parent satisfaction, and postoperative delirium were included. Data synthesis was carried out using Review Manager software (version 5.4). The standardized mean difference (SMD) and 95% confidence intervals (CIs) were used to calculate the effect size for preoperative anxiety. Statistical heterogeneity was assessed with Cochran’s Q test and I^2^ statistics.

**Results:**

A total of 12 studies were included in the meta-analysis, with participants undergoing various types of surgeries. The studies, published between 2017 and 2024, involved children from diverse regions, including South Korea, China, the United States, and others. The results indicated a significant reduction in preoperative anxiety for children who received VR interventions compared to controls (SMD = −0.69, 95% CI [−0.96 to −0.42]). Immersive VR showed a slightly higher effect in attracting children’s attention and alleviating anxiety compared to non-immersive VR. Younger children (aged 3–6) exhibited a higher acceptance of VR and greater anxiety reduction, while older children (aged 7 and above) demonstrated less interest in VR interventions. Anxiety reduction was more pronounced for minor procedures but less effective for complex surgeries. Sensitivity analysis confirmed the robustness of these findings.

**Conclusion:**

VR-based interventions, particularly immersive VR, are effective in reducing preoperative anxiety in pediatric patients undergoing general anesthesia. The efficacy is influenced by the child’s age, the type of surgery, and the specific VR content used. These findings support the clinical application of VR as a tool for anxiety management in pediatric anesthesia settings. While secondary outcomes such as parental anxiety and satisfaction showed positive trends, these findings should be interpreted with caution due to high heterogeneity.

## Introduction

Preoperative anxiety is a prevalent issue affecting 50–75% of pediatric patients. It is significantly associated with adverse postoperative outcomes such as increased pain, prolonged recovery, and emergence delirium (Otto et al., 2025; Perry, Hooper & Masiongale, 2012). High levels of parental anxiety are particularly significant, as they directly influence the child’s distress, creating a cycle of anxiety that negatively impacts the surgical experience (Ryu et al., 2018; Liu et al., 2022; Santapuram et al., 2021).

While pharmacological interventions (such as sedative premedication) and parental presence are commonly used to mitigate this anxiety, they are often limited by potential side effects or inconsistent efficacy (Otto et al., 2025; Liu et al., 2022). Consequently, Virtual Reality (VR) has emerged as a promising non-pharmacological distraction tool. By immersing patients in an interactive environment, VR has demonstrated the potential to alleviate preoperative distress in both children and their parents, thereby improving cooperation during anesthesia induction (Ryu et al., 2018; Santapuram et al., 2021; Barkhordari Ahmadi et al., 2019).

Although several systematic reviews and meta-analyses have investigated the efficacy of VR in pediatric healthcare (Chen, Wang & Chen, 2023; Ahmed et al., 2025; Taylan, Kılıç & Özkan, 2025; Simonetti et al., 2022). current evidence remains insufficient to draw definitive conclusions for specific surgical contexts. Most previous reviews have combined heterogeneous populations, mixing pediatric surgery with dental procedures, burn care, or Venipuncture (Praptidina & Pujiyanto, 2019; Eijlers et al., 2019b). which may obscure the specific effects of VR during general anesthesia induction. Furthermore, many prior meta-analyses included older trials, whereas a significant number of high-quality Randomized Controlled Trials (RCTs) have been published recently. These new studies offer fresh data on secondary outcomes such as parental anxiety and postoperative behavioral changes, which were not fully addressed in earlier reviews (Taylan, Kılıç & Özkan, 2025).

Therefore, the primary objective of this systematic review and meta-analysis was to evaluate the efficacy of VR interventions in reducing preoperative anxiety levels in children undergoing general anesthesia. The secondary objectives were to assess the impact of VR on compliance with anesthesia induction, postoperative pain scores, the incidence of emergence delirium, and parental anxiety levels. Unlike previous works, this review strictly focuses on the preoperative surgical setting to minimize heterogeneity and incorporates the most recent RCTs to provide more robust evidence for clinical decision-making.

## Methods

### Protocol and registry

This systematic review and meta-analysis adhered to the PRISMA guidelines (Page et al., 2021). Additionally, the study protocol was registered on the International Platform for Systematic Reviews and Meta-Analyses Protocol (CRD420251065600).

### Eligibility criteria

#### Inclusion citeria

We applied the PICO framework (Population, Intervention, Comparison, Outcome, and Study) to screen the literature. To be eligible for inclusion, studies had to meet all of the following criteria:

• Population: pediatric patients aged <18 years undergoing elective surgery under general anesthesia. Studies involving adult patients (≥18 years) or patients undergoing procedures under local anesthesia were excluded.

• Intervention: VR-based interventions, which may include both immersive and non-immersive VR. Active interventions involve participants engaging in physical activity, while passive interventions involve participants not engaging in physical activity. VR interventions may be used independently or in combination with other conventional treatments.

• Comparison: Standard treatment, routine care, or placebo control.

• Outcomes: Studies reporting pre-induction anxiety assessed using validated measures (psychometric scales and/or physiological indicators where applicable) and/or relevant secondary outcomes (*e.g.*, compliance with anesthesia induction, postoperative pain, emergence delirium, parental anxiety/satisfaction).

• Study design: Only randomized clinical trials (RCTs), including exploratory RCTs, were considered. Only peer-reviewed articles published in journals, conferences, or seminar papers were included, with no restrictions on language or publication time.

#### Exclusion criteria

Studies were excluded if they met any of the following criteria: duplicate search results; surgeries not involving general anesthesia; study protocol; or studies with unclear outcome measures.

### Search strategy

We conducted a systematic search of the electronic databases PubMed, Embase, and Cochrane Library, with the search period covering from the inception of the databases to 16, February 2026. No additional filtering conditions were applied. The search strategy employed Boolean operators (OR, AND) and was organized according to the key elements of the research question as follows:

Population: (“Anesthesia, General”[Mesh]) OR (General Anesthesia*[Title/Abstract])

Intervention: (“Virtual Reality”[Mesh]) OR ((Virtual Realit*[Title/Abstract]) OR (VR[Title/Abstract])). The retrieval strategies and retrieval results of PubMed, Embase, and Cochrane Library database are shown in Table S1.

### Studies selection

Two independent researchers (SJZ and TYM) screened the titles and abstracts of the retrieved studies, eliminating irrelevant and duplicate entries. The full texts of potentially eligible studies were then reviewed for final inclusion. In cases of disagreement, a third researcher (CQY) resolved the issues.

### Data extraction

Data were extracted from the selected studies using standardized tables. The information extracted included: authors, year, and country; participant characteristics and sample details; intervention type and protocol; measurement scales used; and primary outcomes. Two reviewers (SJZ and RXH) independently performed the data extraction, with a third researcher (BR) participating in the standardization of the extracted data. If necessary, the corresponding authors of the selected studies were contacted to obtain additional information.

### Risk of bias

We have completely re-assessed the risk of bias for all included RCTs strictly using the Cochrane Risk of Bias tool version 2 (RoB 2) (Sterne et al., 2019). We evaluated the five mandatory domains: randomization process, deviations from intended interventions, missing outcome data, measurement of the outcome, selection of the reported result. In cases of disagreement between reviewers, a third researcher (CQY) was consulted to resolve the issues.

### Data analysis

Given the clinical and methodological heterogeneity of the included studies, a random-effects model was employed for the meta-analysis to provide a conservative estimate of the pooled effect. Continuous outcomes measured using the same unit (*e.g.*, heart rate in bpm) were analyzed using the Mean Difference (MD). Heart rate was considered a physiological parameter (proxy marker) rather than an anxiety rating scale and therefore was not pooled within the anxiety-scale subgroup analyses. For outcomes measured using different psychometric anxiety scales (*e.g.*, m-YPAS, STAIC, APAIS, STOA), the Standardized Mean Difference (SMD) was calculated. We used Hedges’ g statistic for SMD calculations to adjust for bias in small sample sizes. Dichotomous outcomes (*e.g.*, incidence of emergence delirium) were analyzed using Risk Ratios (RR). A positive effect size was defined as a situation in which the VR-based intervention resulted in greater reductions in preoperative anxiety compared to the control group. Cochran’s Q test and the I^2^ statistic were used to assess heterogeneity. A *P* value < 0.10 in Cochran’s Q test was considered to indicate statistically significant heterogeneity, while the I^2^ statistic helped to quantify the degree of heterogeneity. If the I^2^ value exceeded 75%, a sensitivity analysis was conducted to test the robustness of the results. The studies were grouped into subgroups based on the measurement scales used for the primary outcome. Data synthesis for this meta-analysis was carried out using Review Manager software (version 5.4; developed by the Cochrane Collaboration).

For studies reporting outcomes at multiple time points, data collected at the time point closest to anesthesia induction (or immediately post-intervention) were selected for the primary analysis. When means and standard deviations (SDs) were not reported, they were derived from other available statistics (*e.g.*, standard errors, confidence intervals) or estimated from medians and interquartile ranges (IQRs) using the methods described by Wan et al. (2014). and the Cochrane Handbook.

Sensitivity analyses were conducted using the “leave-one-out” method to evaluate the stability of the pooled results and to identify individual studies responsible for substantial heterogeneity (I^2^ > 75%). Subgroup analyses were performed based on potential effect modifiers, such as age groups and VR device types.

## Results

### Search results

**Figure 1:**
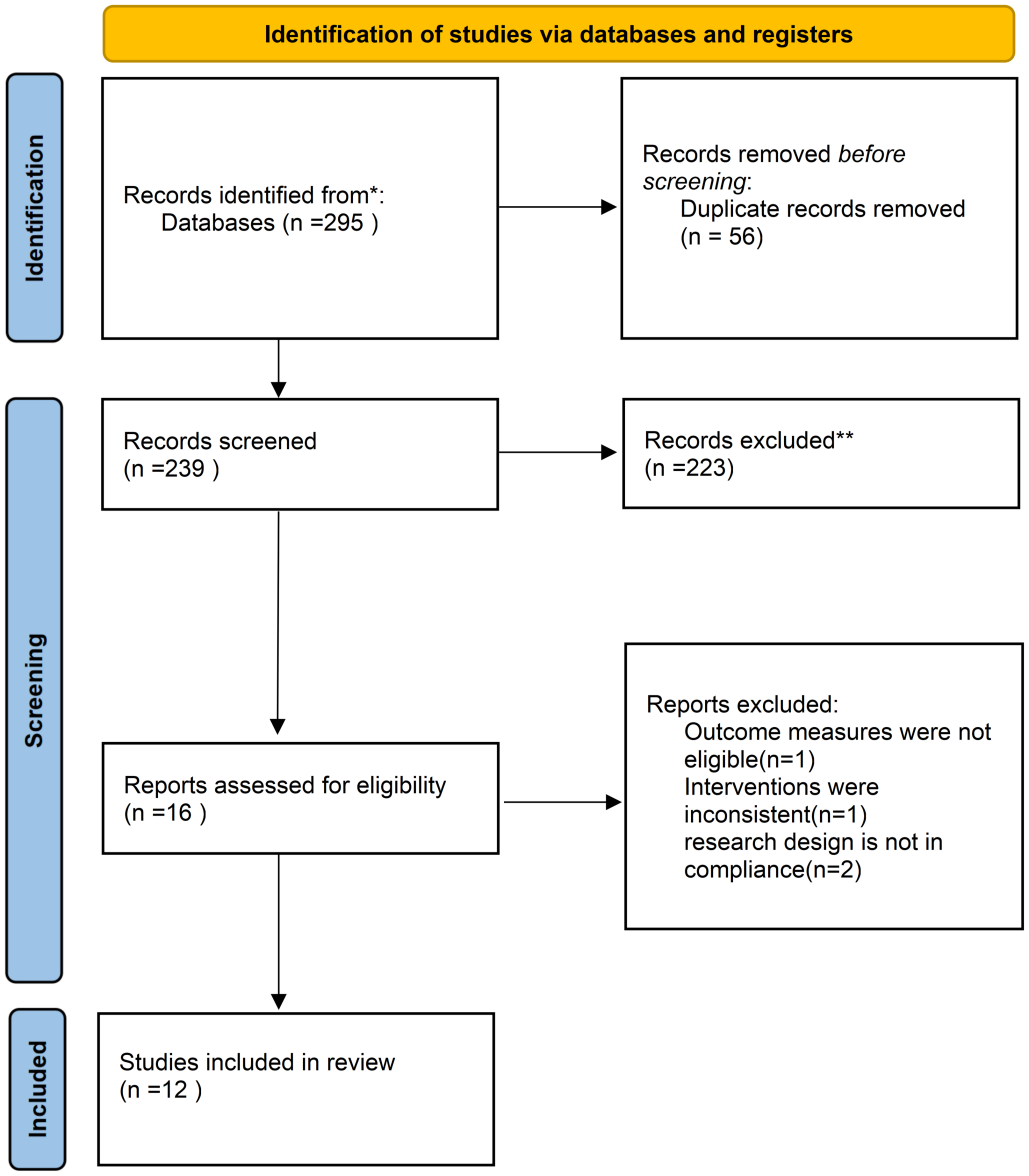
PRISMA 2020 flow diagram of the study selection process.

A total of 295 records were obtained from the comprehensive search. During the preliminary screening process, 56 duplicate records have been deleted., leaving 239 records for further screening. Based on their titles and abstracts, 223 records were excluded, and 16 records were retrieved in full to assess whether they met the inclusion and exclusion criteria. After full-text screening, four records were excluded due to non-compliance with the criteria: one study had outcome measures that did not meet the standards, one had inconsistent interventions, and two had research designs that did not meet the requirements. As a result, 12 records were included for the meta-analysis (Ryu et al., 2017; Ryu et al., 2018; Eijlers et al., 2019a; Park et al., 2019; Ryu et al., 2019; Jung et al., 2021; Vogt et al., 2021; Esposito et al., 2022; Wu et al., 2022; Chiu et al., 2023; Chamberland et al., 2024; Samnakay et al., 2025). The literature screening process is shown in Fig. 1.

### Characteristics of included studies

Among the 12 articles included in this review, four are from South Korea, two from China, one from Italy, one from the Netherlands, one from the United States, one from Germany, one from Australia, and one from Canada. The publication years range from 2017 to 2024, with one article from 2017, one from 2018, three from 2019, two from 2021, two from 2022, one from 2023, and two from 2024. This suggests that the importance of preoperative anxiety in patients undergoing general anesthesia has increasingly gained attention in recent years. Although a wide range of outcomes were explored in the included primary studies, our primary outcome analysis focused on preoperative anxiety, while secondary outcomes were analyzed when available. A complete list of the other outcomes reported in the included studies is provided in Table 1.

### Risk of bias

**Figure 2:**
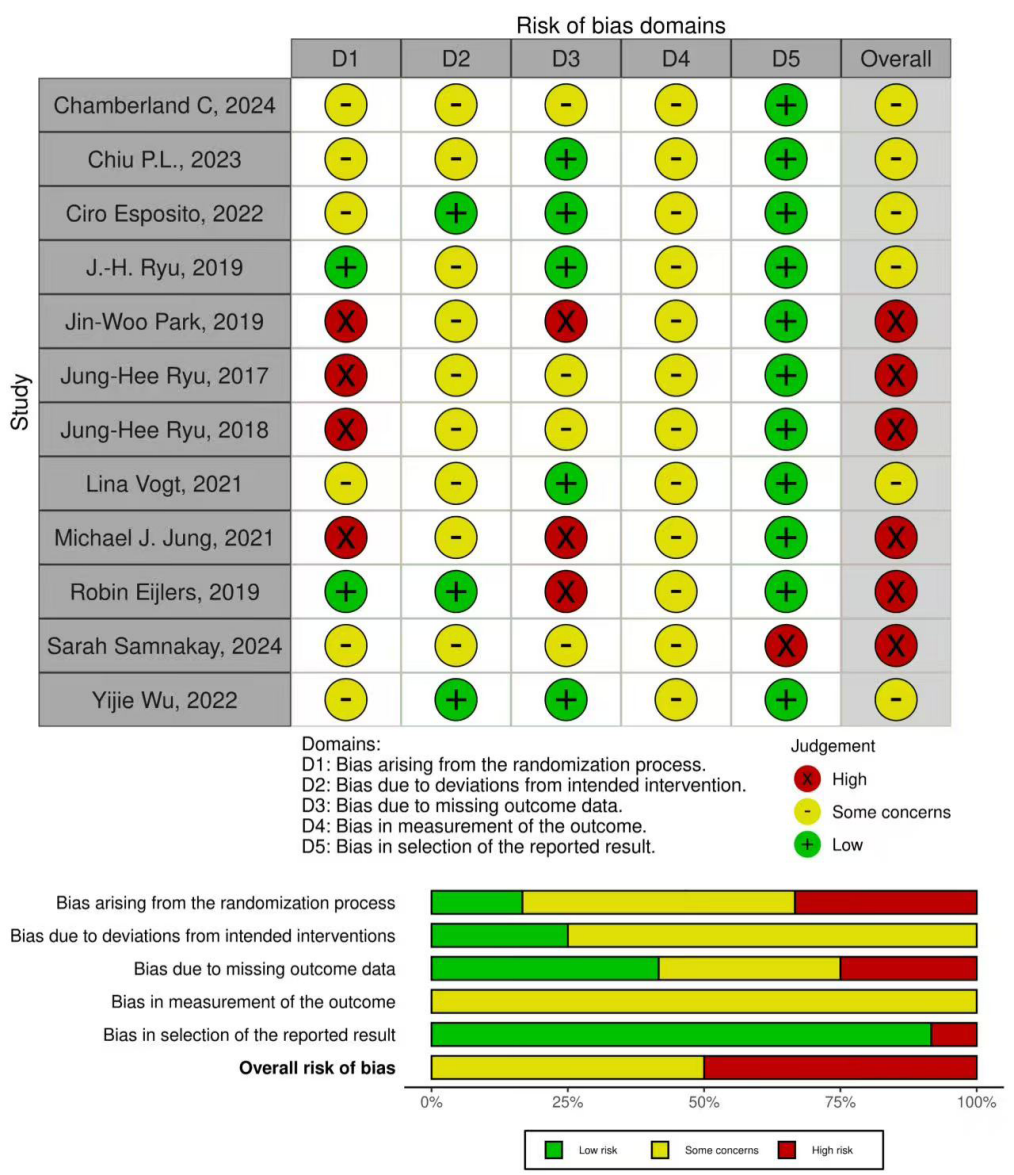
Risk of bias assessment.

The risk of bias for the included randomized controlled trials was evaluated using the Cochrane Risk of Bias 2 tool. The overall assessment revealed that none of the 12 studies were classified as having a low risk of bias, with six studies categorized as high risk and six raising some concerns. In terms of specific domains, the randomization process presented significant limitations as four studies were deemed high risk, six raised some concerns, and two were judged as low risk. Furthermore, deviations from intended interventions raised some concerns in nine studies, largely reflecting the inherent difficulty of blinding participants to virtual reality interventions. While bias due to missing outcome data was present in three high-risk studies, the measurement of the outcome domain was universally flagged with some concerns across all twelve studies due to the lack of assessor blinding. In contrast, the selection of the reported result showed the highest methodological quality, with eleven studies demonstrating a low risk of bias and one study judged as high risk. Detailed bias assessments for each study are presented in Fig. 2 and Table S2.

**Table 1:** The basic characteristics included studies.

**Author**	**Country**	**Year**	**Population**			**Intervention**			**Comparison**			**Outcome measures**	
			**Sample**	**Age**	**Gender**	**Mode of intervention**	**Implementation points**	**Duration**	**Mode of Comparison**	**Implementation points**	**Duration**	**Primary results**	**Secondary outcomes**
Chamberland C	Canada	2024	Control group (n = 64) AR group (n = 37)	Control group: 9 (6–13) AR group: 8 (6–11)	M/F control group:35/29 AR group: 26/11	Augmented reality (AR) glasses	Engage with virtual characters for relaxation exercises and interaction until anesthesia induction	Start 30 min before surgery and until anesthesia induction, lasted about 50.75 min	Receive standard care	Patients receive explanations and reassurance about the procedure without the use of any virtual or augmented reality equipment	Start before surgery, lasted until anesthesia induction	Change in preoperative anxiety	Patient satisfaction, engagement with virtual characters,acceptance of the educational content, postoperative recovery.
Chiu P.L.	Hong Kong, China	2023	Control group: n = 37 VR group: n = 37	Control group: 45.35: [18.00–69.00] VR group: 47.32 [22.00–69.00]	M/F control group:18/19 VR group: 20/17	VR exposure	Wear a VR headset for interactive relaxation exercises and virtual experiences before surgery	Start before surgery, lasted approximately 20 to 30 min	Receive standard care	The patient waits in the preoperative area for routine preoperative information	Start before surgery, lasted during the waiting period until the patient entered the operating room	Change in preoperative anxiety	Preoperative stress, preparedness, postoperative pain, patient satisfaction, length of hospital stay
Robin Eijlers	Amsterdam	2019	Control group: n = 97 VR group: n= 94	Control group: 7.5 [5.6 to 10.7] VR group: 8.3 [5.7 to 10.2]	M/F control group:56/41 VR group: 45/49	VR exposure	Wear a virtual reality headset before surgery and participate in an interactive experience through a guided virtual environment	Start before surgery and until the surgery began,lasted approximately 15 to 20 min	Receive standard care	The patient waits in the preoperative area for routine preoperative information	Start during the preoperative waiting period, until the surgery began.	Postoperative anxiety, pain, and emergence delirium in children	Postoperative analgesic rescue dosage,parental anxiety levels
Ciro Esposito	Italy	2022	Control group: n = 20 VR group: n = 21	Control group: 14 (12–15.5) VR group: 15 (14–17)	M/F control group: 12 /8 VR group: 13 /7	VR exposure	Wear a VR headset before surgery and engage in a relaxing virtual reality game	Start before surgery until the child entered the operating room, lasted approximately 30 min	Receive standard care	The patient waits in the preoperative area for routine preoperative information	Start during the preoperative waiting period, until entering the operating room	Preoperative anxiety levels, assessed primarily by heart rate, facial expression scores.	Children’s subjective ratings of the virtual reality experience, anesthesiologist’s observational ratings
Michael J. Jung	California	2021	Control group: n = 37 VR group: n = 33	Control group: 7.8 ± 2.3 VR group: 8.2 ± 2.2	M/F control group: 19 /18 VR group: 15 /18	VR exposure	Children wore VR headsets and engaged in immersive virtual reality experiences	Start before anesthesia induction, lasted approximately 15 to 20 min	Used other distraction methods, such as wearing headphones,	Control group wore headphones and engaged in audio or other distraction activities	Start before anesthesia induction, laste approximately 15 to 20 min	The change in preoperative anxiety	Post-induction parental anxiety, pediatric induction compliance, parental satisfaction.
Jin-Woo Park	Korea	2019	Control group: n = 40 VR group: n = 40	Control group: 6.75 (5.3–8.8) VR group: 7.1 (5.8–8.4)	M/F control group: 20/20 VR group: 27 /13	VR exposure	The child watches the VR video tour through a smartphone and VR headset, and the parent watches using a mirrored display device and discusses the preoperative process with the child	Start 1 h prior to surgery, lasted approximately 15 to 20 min	Experienced the virtual reality (VR) tour of the operating room	Children watched the VR tour of the operating room using a VR headset, without the parents’ participation in the viewing process	Start 1 h prior to surgery, lasted approximately 15 to 20 min	The change in children’s preoperative anxiety	Children’s anesthesia compliance parents/guardians’ preoperative anxiety, satisfaction with the overall anesthesia preparation process
Jung-Hee Ryu	Korea	2017	Control group: n = 39 VR group: n = 41	Control group: 6 (5–8) VR group: 6 (5–7)	M/F control group: 21/18 VR group: 29/12	VR exposure	The child watches the video tour through a VR headset, and the parent watches using a mirrored display device and discusses the preoperative procedure with the child	Start 1 h prior to surgery, lasted approximately 4 min	Received traditional preoperative education, including text and pictures	Children and parents are informed about the operating room and surgical process through text and pictures, with parents accompanying the children throughout	Start 1 h prior to surgery, lasted approximately 4 min	The change in children’s preoperative anxiety	Incidence and severity of emergence delirium, postoperative behavioral disturbances
Jung-Hee Ryu	Korea	2018	Control group: n = 35 VR group: n = 34	Control group: 6 (5–8) VR group: 5 (5–7)	M/F Control Group: 22/13 VR group: 18/16	VR exposure	Children participate and experience through VR games	Start before the child receive general anesthesia until anesthesia induction	Received conventional preoperative education	It is carried out through traditional educational methods without the use of VR technology	Start before the child receive general anesthesia until anesthesia induction	The change in preoperative anxiety	Compliance during anesthesia induction, preoperative behavioral performance, parental satisfaction scores.
Sarah Samnakay	Australia	2024	3D VR: n = 98 2D video: n = 90	3D VR: 8.83 ± 2.8 2D video: 8.80 ± 2.9	M/F 3D VR: 60/38 2D video: 52/38	3D VR exposure and 2D videos	Children intervene by using 3D VR and 2D video to distract themselves	Start before child enters the preoperative preparation room and until anesthesia induction	2D videos	The control group used 2D video as the object and watched the same video content	Start before child enters the preoperative preparation room and until anesthesia induction	Changes in anxiety levels in children in the preoperative preparation area and anesthesia induction phase	State anxiety in children, The child’s cooperation during anesthesia induction
Lina Vogt	Germany	2021	Control group: n = 34 VR group: n = 34	72 patient: 54.19 (20–81)	M/F: 35/37	Virtual Operating Room Tour (VORT)	The operating room environment is introduced using the V R to help patients become familiar with the surgical process	Start 1 h before surgery, lasted approximately 15 min	2D video tour	A 2D video tour is used for preoperative preparation and provides basic information about the operating room environment	Start 1 h before surgery, lasted approximately 15 min	The effect on preoperative anxiety	Overall patient rating of VORT, satisfaction with preoperative preparation, the reduction of preoperative anxiety
Yijie Wu	China	2022	Control group: n = 48 VR group: n = 51	Control group: 8.0 (5.0–9.75) VR group: 7.0 (6.0–9.0)	M/F Control Group: 42/6 VR group: 44/7	VR exposure	Parents and children watch VR videos together that show the process before and after surgery, and encourage parents to help their children recall the video content	Start before surgery, lasted approximately 15 to 20 min	Receive standard care	The health care provider informs the child about the surgery, but does not provide an immersive experience	Start before surgery, lasted approximately 15 to 20 min	Anxiety experienced by children during anesthesia induction	Children’s compliance during induction, postoperative pain, emergence delirium, parental anxiety levels, parental satisfaction
J.-H. Ryu	Korea	2019	Control group: n = 35 VR group: n = 34	Control group: 6 (5–9) VR group: 6 (5–7)	M/F Control Group: 24/11 VR group: 17/17	VR exposure	Use smartphones and VR headsets to help children familiarize themselves with the operating room environment	Provided once before the surgery	Through videos or pamphlets	Children and parents are introduced to the operating room environment and procedures through videos or booklets	Provided once before the surgery	Preoperative anxiety	Compliance during anaesthesia induction, procedural behaviour rating scale

### Primary outcome measures

**Figure 3:**
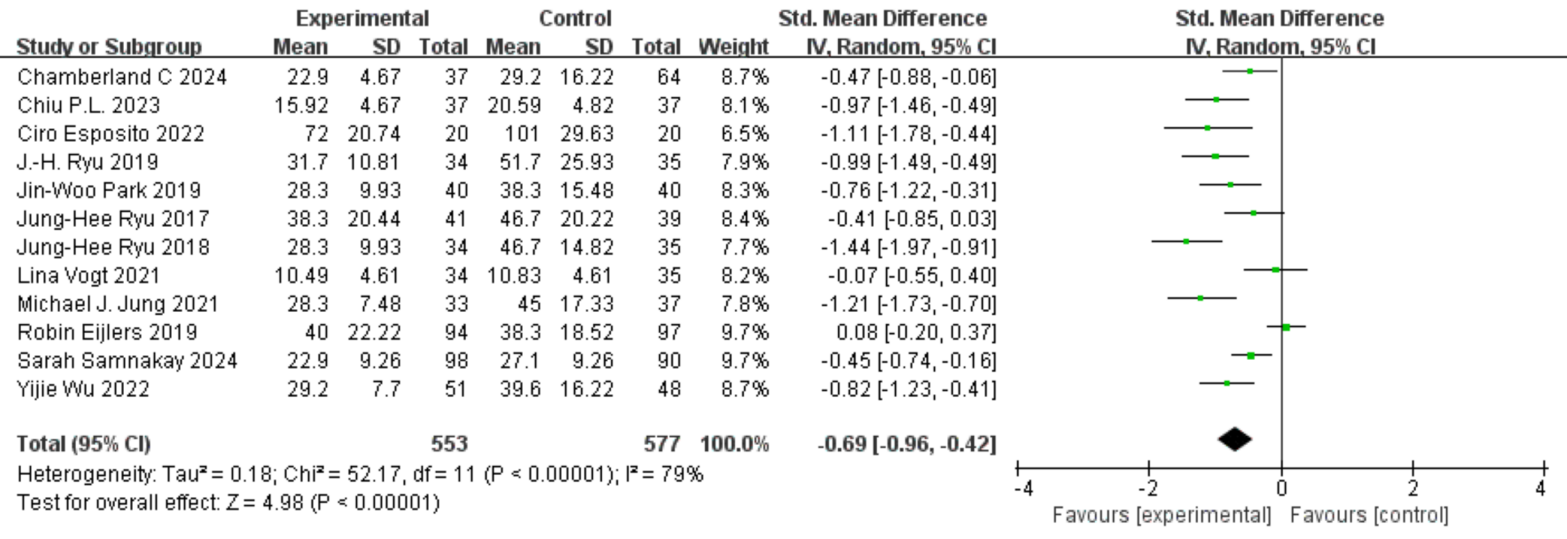
Forest plot of pre-anesthesia anxiety.

**Figure 4:**
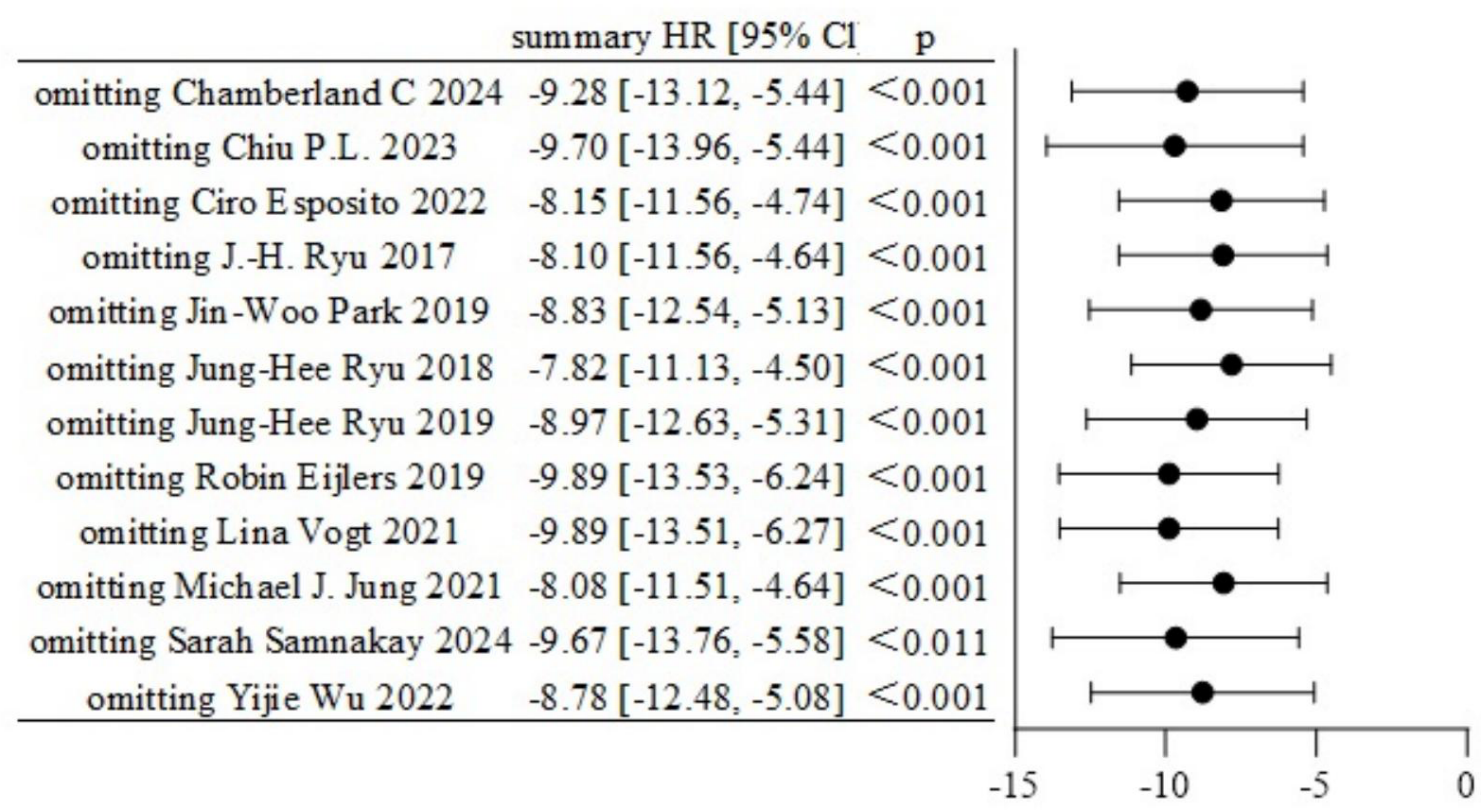
Sensitivity analysis of pre-anesthesia anxiety.

**Figure 5:**
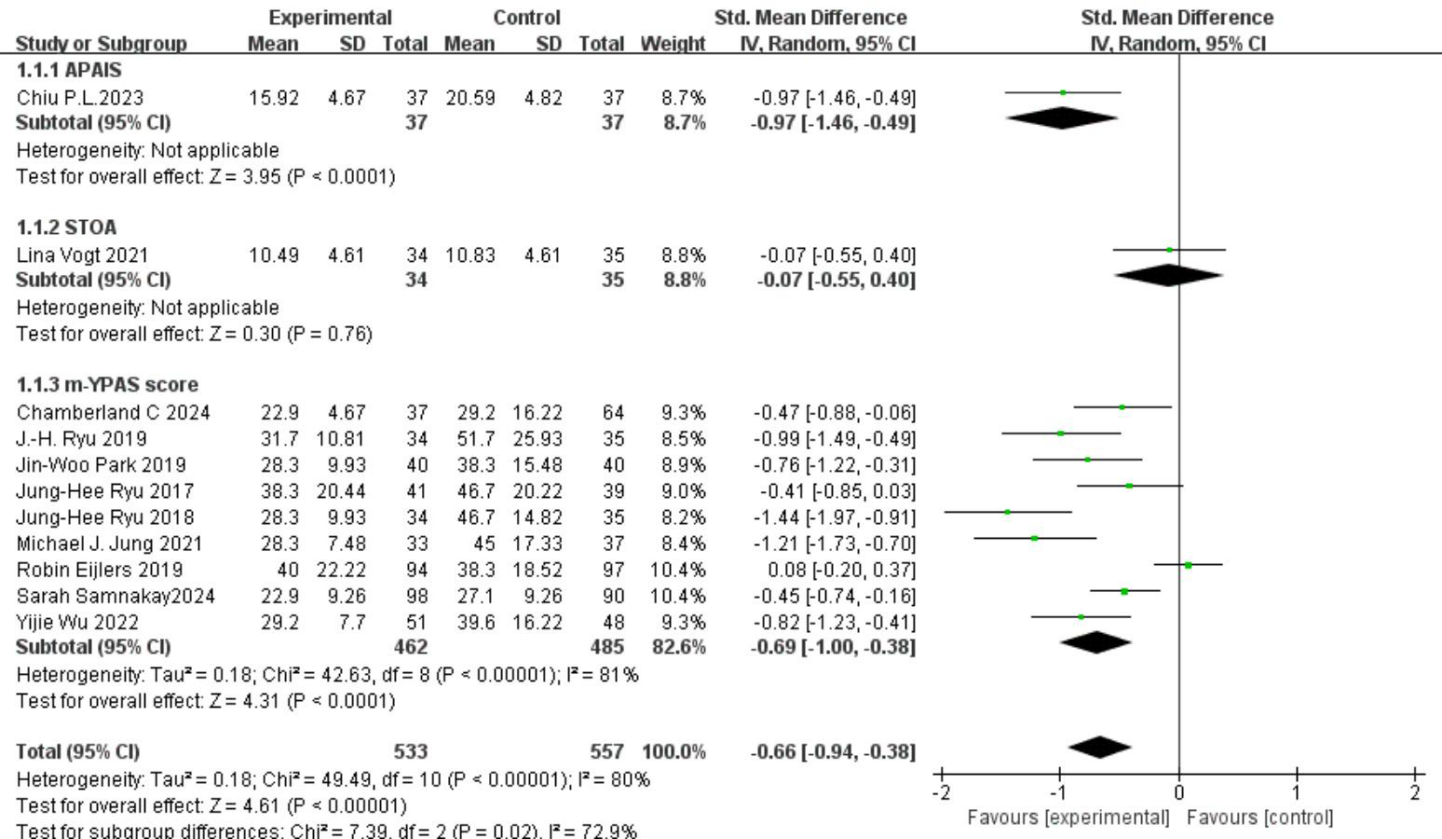
Subgroup analysis forest plot of pre-anesthesia anxiety in children.

Twelve studies reported data on pre-anesthesia anxiety, involving a total of 1,130 participants, with 553 in the experimental group and 577 in the control group (Ryu et al., 2018; Liu et al., 2022; Santapuram et al., 2021; Barkhordari Ahmadi et al., 2019; Chen, Wang & Chen, 2023; Ahmed et al., 2025; Taylan, Kılıç & Özkan, 2025; Simonetti et al., 2022; Praptidina & Pujiyanto, 2019; Eijlers et al., 2019b; Page et al., 2021; Sterne et al., 2019). There was substantial heterogeneity among the studies (I^2^ = 87%, *P* < 0.0001). A random-effects model was applied, and the analysis showed that the experimental group had significantly lower anxiety scores than the control group (SMD = −0.69, 95% CI [−0.96, −0.42]), as shown in Fig. 3. Sensitivity analysis using the leave-one-out method confirmed the stability of the results (Fig. 4). Subgroup analysis based on psychometric anxiety scales revealed that, compared to the control group, the experimental group showed significant reductions in pre-anesthesia anxiety as measured by the APAIS scale (SMD = −0.97, 95% CI [−1.46 to −0.49]), the STOA scale (SMD = −0.07, 95% CI [−0.55 to 0.40]), and the m-YPAS scale (SMD = −0.69, 95% CI [−1.00 to −0.38]), as shown in Fig. 5. Heart rate was treated as a secondary physiological outcome and was summarized separately rather than being grouped under anxiety rating scales. One study reported pre-induction heart rate; as a physiological outcome, it was summarized descriptively (VR *vs* control: 72 ± 20.74 bpm *vs* 101 ± 29.63 bpm; MD −29.0 bpm, 95% CI [−44.85 to −13.15]).

### Parental satisfaction

**Figure 6:**
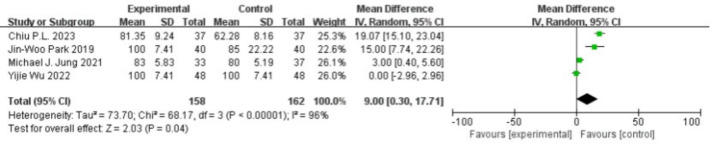
Forest plot of parental satisfaction.

Four studies reported parental satisfaction, involving a total of 320 participants, with 158 in the experimental group and 162 in the control group (Chen, Wang & Chen, 2023; Ahmed et al., 2025; Praptidina & Pujiyanto, 2019; Eijlers et al., 2019b). There was substantial heterogeneity among the studies (*P* < 0.00001, I^2^ = 96%), and a random-effects model was used. The analysis showed that the difference in parental satisfaction scores between the two groups were significantly higher (MD = 9.00, 95% CI [0.30–17.71], *P* = 0.04), as shown in Fig. 6.

### Parental anxiety after induction

**Figure 7:**
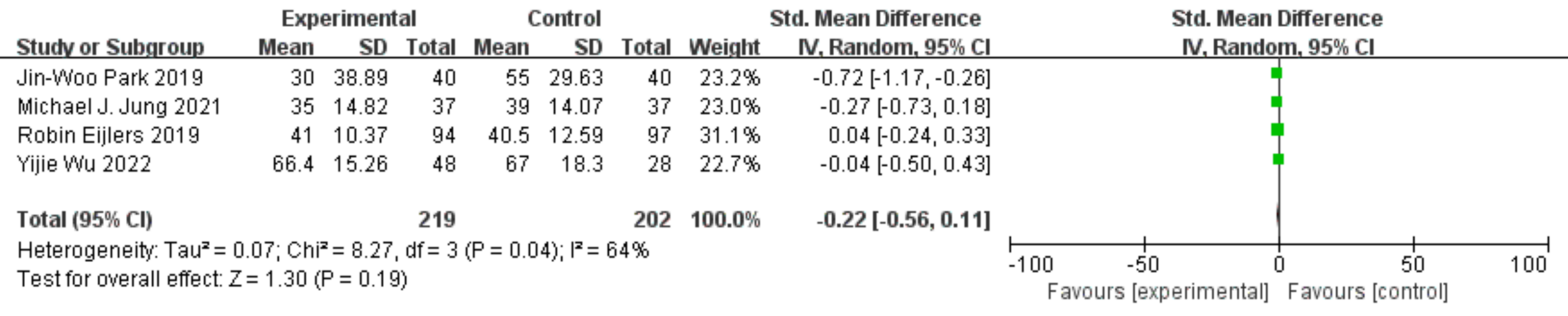
Forest plot of parental anxiety status after induction.

Four studies reported data on parental anxiety after induction, with a total of 421 participants, including 219 in the experimental group and 202 in the control group (Santapuram et al., 2021; Barkhordari Ahmadi et al., 2019; Ahmed et al., 2025; Praptidina & Pujiyanto, 2019). There was substantial heterogeneity among the studies (*P* = 0.01, I^2^ = 73%), and a random-effects model was applied. The results indicated that the difference in parental anxiety scores between the two groups was not statistically significant [SMD = −0.22, 95% CI [−0.56 to 0.11], *P* = 0.19], as shown in Fig. 7.

### Postoperative delirium severity

**Figure 8:**
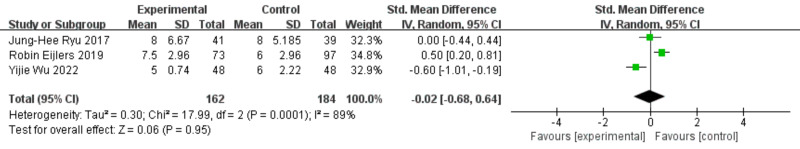
Forest plot of the incidence of delirium in patients after surgery.

Three studies reported on the severity of postoperative delirium, involving a total of 346 participants, with 162 in the experimental group and 184 in the control group (Santapuram et al., 2021; Chen, Wang & Chen, 2023; Praptidina & Pujiyanto, 2019). There was substantial heterogeneity among the studies (*P* < 0.00001, I^2^ = 90%), and a random-effects model was used. The analysis showed that the difference in delirium severity scores between the two groups was not statistically significant (SMD = −0.02, 95% CI [−0.68 to 0.64], *P* = 0.95), as shown in Fig. 8.

## Discussion

Recent meta-analyses and systematic reviews have consistently demonstrated the efficacy of VR interventions in reducing preoperative anxiety. A recent 35-item randomized controlled trial analysis looked at the main components of virtual reality interventions (*i.e.,* device, medium, format and duration) ,Patients undergoing elective surgery with anesthesia may benefit from VR as a novel alternative to reduce preoperative anxiety, especially pediatric patients *via* the distraction approach (Li et al., 2025). A meta-analysis of 10 RCTs found that VR significantly lowered preoperative anxiety, particularly in pediatric patients (Koo et al., 2020). However, a systematic review focusing on adult patients reported inconsistent findings, suggesting the need for larger, high-quality RCTs to definitively determine VR’s effectiveness in reducing perioperative anxiety among adults (Asiri et al., 2025). This meta-analysis showed that patients who received the VR intervention had significantly improved preoperative anxiety levels compared to those who received conventional care. This suggests that VR technology, as an adjunctive intervention, can effectively alleviate preoperative anxiety, especially in patients undergoing general anesthesia, and has broad clinical potential.

Recent studies have verified the efficacy of virtual reality (VR) in alleviating pain and anxiety during medical procedures, with particularly pronounced benefits in pediatric and adolescent populations.Whether it is immersive or non-immersive VR mode, immersive VR usually shows better effects (Kumari et al., 2021). Specifically, immersive VR can markedly reduce preoperative anxiety and fear in adult patients (Flores et al., 2023) and effectively manage pain and distress in pediatric settings (Sánchez-Caballero, Ortega-Donaire & Sanz-Martos, 2024). with its analgesic and anxiolytic effects primarily attributed to the distraction mechanism—diverting patients’ attention away from painful or anxiety-provoking stimuli (Indovina et al., 2018).This study compared the effectiveness of immersive VR and non-immersive VR. Immersive VR typically offers a more engaging, interactive experience, allowing patients to fully immerse themselves in a virtual environment (Hao et al., 2023). In contrast, Non-immersive VR uses screen-displayed content; immersive VR’s superior interactivity and sensory stimulation better reduce surgical anxiety *via* distraction. Non-immersive VR, though less engaging, remains beneficial for resource-limited or simplified interventions.

This study found that the type of VR content used also influenced the intervention’s effectiveness. VR incorporating animation and gamified elements proved more effective at capturing patient’s attention and alleviating anxiety than static scenario presentations or video content. The interactive, game-like experience provided a more engaging and enjoyable atmosphere, which helped patients manage their fear of surgery. On the other hand, simple scenario-based VR presentations yielded comparatively weaker effects. In addition,VR’s efficacy may vary depending on the type of procedure, with greater benefits observed in dental studies and when using non-interactive VR (Smith, Wang & Colloca, 2022). Despite promising results, challenges remain, including high equipment costs and limited research with small sample sizes (Smith, Wang & Colloca, 2022; Domene et al., 2025). VR interventions reduced anxiety significantly in patients with mild, non-invasive outpatient surgeries, but showed weaker effects for major complex procedures (*e.g.*, thoracic/abdominal surgeries), probably owing to the greater psychological burden of these surgeries.

In this meta-analysis, we reviewed data on parental satisfaction, anxiety after induction, and postoperative delirium. Some studies have found no significant difference in parental anxiety between groups with and without parental presence (Blesch & Fisher, 1996; Erhaze, Dowling & Devane, 2016), others report that parents and children experience reduced anxiety when induced (Shih et al., 2023). Studies have found that audio-visual interventions have a modest positive effect on both parents and children’s preoperative anxiety (Chow et al., 2018). Parental presence may increase parental satisfaction and will not hinder operating room efficiency (Shih et al., 2023). However, the clinical significance of these findings remains unclear, and high variability between studies remains (Erhaze, Dowling & Devane, 2016). Despite conflicting evidence, 98.03% of parents wished to be present for follow-up surgery (Shih et al., 2023). Further research on larger, more consistent samples is needed to draw more definitive conclusions. Despite the large number of studies and participants, the results showed no significant differences between the experimental and control groups. Although there was a slight improvement in parental satisfaction and anxiety in the experimental group, the overall effect was small.

The difference in postoperative delirium incidence was almost negligible. These findings suggest that the analyzed interventions may not significantly improve parents’ mood or symptoms, and the high variability between studies could be due to differences in study design, interventions, or participant characteristics. Given the considerable heterogeneity, the pooled estimates for parental anxiety and satisfaction must be viewed as exploratory. The high variability likely stems from distinct cultural attitudes toward surgery, differing anxiety scales, and variations in VR protocols. Therefore, while the current evidence does not support a strong intervention effect, future studies with larger, more consistent samples may provide clearer conclusions.

Despite following rigorous methodological standards, there are several limitations that may affect the accuracy and generalizability of these findings. Firstly, the studies included in this analysis exhibited substantial heterogeneity (I^2^ = 96%) (Imrey, 2020), likely due to variations in participant characteristics, the content of interventions, and the implementation methods. Although a random-effects model was applied to address this issue, the remaining heterogeneity may still compromise the consistency and stability of the results. Secondly, the uneven allocation of participants between intervention and control groups in some studies may introduce selection bias, influencing the interpretation of the findings. Additionally, while this meta-analysis included multiple studies, some had small sample sizes, which may result in low statistical power and limit the generalizability of the findings (Imrey, 2020). Lastly, certain studies failed to provide complete clinical data or contained methodological flaws, which could introduce bias into the results (Ioannidis & Lau, 1999).

## Conclusions

VR is an effective intervention for reducing preoperative anxiety in children, outperforming traditional treatments, which offers a valuable non-pharmacological approach for preparing children for surgery by easing anxiety and enhancing cooperation. As technology advances, VR has significant potential in clinical practice, particularly for managing preoperative anxiety in children.

## Supplemental Information

10.7717/peerj.21123/supp-1 Supplemental Information 1 PRISMA checklist

10.7717/peerj.21123/supp-2 Supplemental Information 2 The retrieval strategies and retrieval results of each database

10.7717/peerj.21123/supp-3 Supplemental Information 3 The detailed materials for the risk assessment of bias

## Additional Information and Declarations

### Data Availability

The following information was supplied regarding data availability: The data is available at Mendeley: song, jinzhuo (2026), “Efficacy of virtual reality interventions in reducing preoperative anxiety in pediatric patients undergoing general anesthesia: a systematic review and meta-analysis”, Mendeley Data, V1, doi: 10.17632/8f4mmgr2fg.1.
