# Supplementary material for: Efficacy of virtual reality interventions in reducing preoperative anxiety in pediatric patients undergoing general anesthesia: a systematic review and meta-analysis
Source: PeerJ. 2026 Apr 22;14:e21123. doi: 10.7717/peerj.21123 (PMC13109977; doi:10.7717/peerj.21123)
Supplement: Supplemental Information 2 [file peerj-14-21123-s002.docx]

**Table S1：The retrieval strategies and retrieval results of each database**

PubMed

| No. | Content | Result |
| --- | --- | --- |
| #1 | Search: "Virtual Reality"[Mesh] Sort by: Most Recent | 10047 |
| #2 | Search: (Virtual Realit*[Title/Abstract]) OR (VR[Title/Abstract]) Sort by: Most Recent | 33609 |
| #3 | Search: ("Virtual Reality"[Mesh]) OR ((Virtual Realit*[Title/Abstract]) OR (VR[Title/Abstract])) Sort by: Most Recent | 35151 |
| #4 | Search: "Anesthesia, General"[Mesh] Sort by: Most Recent | 64578 |
| #5 | Search: General Anesthesia*[Title/Abstract] Sort by: Most Recent | 53657 |
| #6 | Search: ("Anesthesia, General"[Mesh]) OR (General Anesthesia*[Title/Abstract]) Sort by: Most Recent | 101273 |
| #7 | Search: "Anxiety"[Mesh] Sort by: Most Recent | 133434 |
| #8 | Search: ((((Angst[Title/Abstract]) OR (Nervousness[Title/Abstract])) OR (Hypervigilance[Title/Abstract])) OR (Anxiet*[Title/Abstract])) OR (Anxiousness[Title/Abstract]) Sort by: Most Recent | 316446 |
| #9 | Search: ("Anxiety"[Mesh]) OR (((((Angst[Title/Abstract]) OR (Nervousness[Title/Abstract])) OR (Hypervigilance[Title/Abstract])) OR (Anxiet*[Title/Abstract])) OR (Anxiousness[Title/Abstract])) Sort by: Most Recent | 338898 |
| #10 | Search: ((("Virtual Reality"[Mesh]) OR ((Virtual Realit*[Title/Abstract]) OR (VR[Title/Abstract]))) AND (("Anxiety"[Mesh]) OR (((((Angst[Title/Abstract]) OR (Nervousness[Title/Abstract])) OR (Hypervigilance[Title/Abstract])) OR (Anxiet*[Title/Abstract])) OR (Anxiousness[Title/Abstract])))) AND (("Anesthesia, General"[Mesh]) OR (General Anesthesia*[Title/Abstract])) Sort by: Most Recent | 34 |

Embase

| No. | Content | Result |
| --- | --- | --- |
| #1 | virtual AND ('reality'/exp OR reality) | 53752 |
| #2 | 'virtual realit*':ab,ti OR 'vr':ab,ti | 44322 |
| #3 | #1 OR #2 | 65118 |
| #4 | anesthesia, AND general | 223199 |
| #5 | 'general anesthesia*':ab,ti | 84357 |
| #6 | #4 OR #5 | 223211 |
| #7 | anxiety | 661043 |
| #8 | 'angst':ab,ti OR 'nervousness':ab,ti OR 'hypervigilance':ab,ti OR 'anxiet*':ab,ti OR 'anxiousness':ab,ti | 488287 |
| #9 | #7 OR #8 | 666759 |
| #10 | #3 AND #6 AND #9 | 165 |

Cochrane Library

| No. | Content | Result |
| --- | --- | --- |
| #1 | Virtual Reality | 9881 |
| #2 | (Virtual Realit*):ab,ti,kw OR (VR):ab,ti,kw | 10990 |
| #3 | #1 or #2 | 11211 |
| #4 | Anesthesia, General | 43770 |
| #5 | (General Anesthesia*):ab,ti,kw | 34956 |
| #6 | #4 or #5 | 43783 |
| #7 | anxiety | 98494 |
| #8 | (Angst):ab,ti,kw OR (Nervousness):ab,ti,kw OR (Hypervigilance):ab,ti,kw OR (Anxiet*):ab,ti,kw OR (Anxiousness):ab,ti,kw | 93619 |
| #9 | #7 or #8 | 96385 |
| #10 | #3 AND #6 AND #9 | 117 |
