# Supplementary material for: Efficacy of virtual reality interventions in reducing preoperative anxiety in pediatric patients undergoing general anesthesia: a systematic review and meta-analysis
Source: PeerJ. 2026 Apr 22;14:e21123. doi: 10.7717/peerj.21123 (PMC13109977; doi:10.7717/peerj.21123)
Supplement: Supplemental Information 3 [file peerj-14-21123-s003.docx]

**Table S2：The detailed materials for the risk assessment of bias.**

|  | D1 | D2 | D3 | D4 | D5 |
| --- | --- | --- | --- | --- | --- |
| Chamberland C, 2024 | Some concerns | Some concerns | Some concerns | Some concerns | Low |
| Chiu P.L., 2023 | Some concerns | Some concerns | Low | Some concerns | Low |
| Ciro Esposito, 2022 | Some concerns | Low | Low | Some concerns | Low |
| J.-H. Ryu, 2019 | Low | Some concerns | Low | Some concerns | Low |
| Jin-Woo Park, 2019 | High | Some concerns | High | Some concerns | Low |
| Jung-Hee Ryu, 2017 | High | Some concerns | Some concerns | Some concerns | Low |
| Jung-Hee Ryu, 2018 | High | Some concerns | Some concerns | Some concerns | Low |
| Lina Vogt, 2021 | Some concerns | Some concerns | Low | Some concerns | Low |
| Michael J. Jung, 2021 | High | Some concerns | High | Some concerns | Low |
| Robin Eijlers, 2019 | Low | Low | High | Some concerns | Low |
| Sarah Samnakay, 2024 | Some concerns | Some concerns | Some concerns | Some concerns | High |
| Yijie Wu, 2022 | Some concerns | Low | Low | Some concerns | Low |
